# Complications in Patients Undergoing Laparoscopic Bariatric Surgery in an ERABS-optimized, High-Volume, Single Center During 2020 and 2021

**DOI:** 10.1007/s11695-023-06596-1

**Published:** 2023-05-18

**Authors:** Katrine Stryhn, Lærke Alstrup, Claus Riber, Michael Ørting, Rakin Hadad, Jan Hvistendahl, Carsten Tollund, Niels Boye, Steen B. Haugaard, Peter Funch-Jensen

**Affiliations:** 1 Department of Surgery, Aleris Hospitals, Copenhagen Department, Gyngemose Parkvej 66, 2860 Søborg, Denmark; 2 Institute of Clinical Medicine, Faculty of Health and Medical Science, University of Copenhagen, Blegdamsvej 3B, 2200 Copenhagen, Denmark; 3 Department of Surgery, Aleris Hospitals, Aarhus Department, Brendstrupgårdsvej 21, 8200 Aarhus, Denmark; 4 Department of Surgery, Aleris Hospitals, Esbjerg Department, Bavnehøjvej 2, 6700 Esbjerg, Denmark; 5 Department of Cardiology, Bispebjerg University Hospital, Bispebjerg Bakke 23, 2400 Copenhagen, Denmark; 6 Department of Anesthesiology, Aleris Hospitals, Aarhus Department, Brendstrupgårdsvej 21, 8200 Aarhus, Denmark; 7 Department of Anesthesiology, Aleris Hospitals, Esbjerg Department, Bavnehøjvej 2, 6700 Esbjerg, Denmark; 8 Department of Anesthesiology, Aleris Hospitals, Copenhagen Department, Gyngemose Parkvej 66, 2860 Søborg, Denmark; 9 Department of Endocrinology, Aleris Hospitals, Copenhagen Department, Gyngemose Parkvej 66, 2860 Søborg, Denmark; 10 Department of Endocrinology, Aleris Hospitals, Aarhus Department, Brendstrupgårdsvej 21, 8200 Aarhus, Denmark; 11 Department of Endocrinology, Aleris Hospitals, Esbjerg Department, Bavnehøjvej 2, 6700 Esbjerg, Denmark; 12 Department of Endocrinology, Bispebjerg University Hospital, Bispebjerg Bakke 23, 2400 Copenhagen, Denmark; 13 Department of Clinical Medicine, Aarhus University, Palle Juul-Jensens Boulevard 82, 8200 Aarhus, Denmark

**Keywords:** Bariatric surgery, Sleeve gastrectomy, Fast-track surgery, ERAS, ERABS, Clavien-Dindo classification

## Abstract

**Purpose:**

Complication rates after fast-track optimization in bariatric surgery are varying. The aim of this study was to identify short-term complications in patients undergoing laparoscopic sleeve gastrectomy (SG) in an ERABS (enhanced recovery after bariatric surgery) optimized setup.

**Materials and Methods:**

This study is an observational analysis of a consecutive cohort of 1600 patients undergoing SG at an ERABS-optimized, private hospital during 2020 and 2021. Primary outcomes were length of stay, mortality, readmissions, reoperations, and complications according to the Clavien-Dindo classification (CDC) within postoperative day (POD) 30 and 90. Secondary outcomes were weight loss and quality of life (QoL) according to Moorehead-Ardelt questionnaires during the first postoperative year.

**Results:**

**Primary outcomes: **99.1% of patients were discharged within POD 1. The 90-day mortality rate was zero. There were 1% readmissions and 1.2% reoperations within POD 30. Total 30-day complication rate was 4.6%, where 3.4% accounted for CDC grades ≤ II, and 1.3% accounted for CDC grade III. There were zero grade IV–V complications. **Secondary outcomes:** One year after surgery, weight loss was substantial (*p* < 0.001), with an excess weight loss of 71.9%, and QoL had significantly increased (*p* < 0.001).

**Conclusion:**

This study demonstrates that the use of an ERABS protocol in bariatric surgery does not compromise neither safety nor efficacy. Complication rates were low, and weight loss was significant. This study thus provides strong arguments that ERABS programs are beneficial in bariatric surgery.

**Graphical Abstract:**

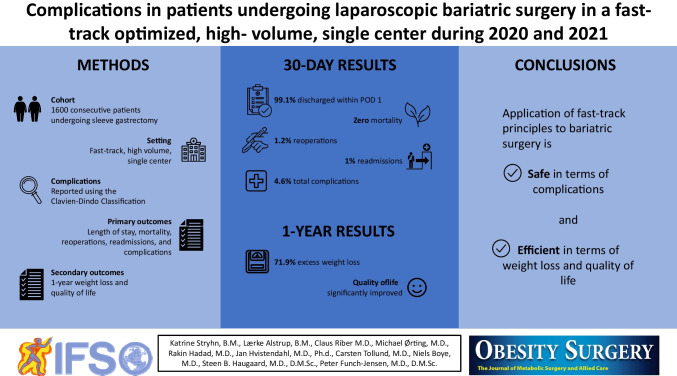

## Introduction

Fast-track principles have revolutionized the surgical field by optimizing outcomes of surgery in terms of lowering overall morbidity as well as length of hospital stay. The approach is widely implemented in numerous surgical fields with significant outcome improvement [1, 2]. In the bariatric field, however, implementation of ERABS (enhanced recovery after bariatric surgery) programs have been reported to cause both lower, identical, and higher complication rates as compared to conventional care [3–7].

These conflicting results may partly be due to a lack of consistency in the reporting of surgical complications. By the development of the Clavien-Dindo classification (CDC), a standardized tool for describing surgical complications was offered, allowing for better comparison between studies [8, 9]. CDC has been widely accepted in the surgical literature since its proposal and evaluation in 2004 and 2009, respectively [8–10].

The aim of our study was to analyze complications in a large cohort of consecutive patients undergoing sleeve gastrectomy (SG) in an ERABS-optimized, high-volume, single center. We used CDC to quantify complications in an objective and comparable manner.

## Materials and Methods

### Study Cohort and Setting

All patients undergoing SG as a primary bariatric surgery at Aleris Hospitals in Denmark during 2020 and 2021 were included. The operations were performed laparoscopically by two highly experienced surgeons (> 3000 bariatric procedures each). No operations were converted to open surgery.

Aleris is a private hospital chain with three hospitals performing bariatric surgery. The patient course and protocol were identical at the three settings, and the health care professionals involved interchanged between hospital units.

Indication for surgery followed international guidelines [11]. Patients with BMI (body mass index) ≥ 30 kg/m^2^ could meet criteria for surgery based upon individual assessment. Prior to surgery, a surgical, medical, and dietary record was obtained by an endocrinologist, a surgeon, and a dietitian, respectively. Patients were referred to other specialists for further investigation if necessary. Preoperative weight loss was recommended on an individual basis.

The patient course met the requirements of the most recent ERABS guidelines, except for preoperative carbohydrate loading and postoperative gallstone prevention with ursodeoxycholic acid [12]. Perioperative neuromuscular blockage was not used, however, in some cases, a short blockage was induced during intubation.

### Perioperative Course

Premedication consisted of peroral paracetamol 1 g, etodolac 400 mg, gabapentin 600 mg, and intravenous (i.v.) dexamethasone 8 mg. Anesthesia was induced and maintained using i.v. remifentanil and propofol. Succinylcholine was used prior to intubation if necessary. Peroperatively, 1000-ml isotonic saline and 1.5-g cefuroxime were administered i.v. Prior to termination of surgery, 100 μg fentanyl, 0.2 g dehydrobenzperidol, 4 mg ondansetron, and 1 mg clemastin were administered i.v.

### Surgical Technique

The operations were performed by a standardized 5-port surgical technique, using a disposable surgical kit from Medtronic, MN, USA. Prior to insertion of the Veress cannula, 40 cc 0.5% Marcaine were injected at the port sites.

The surgery was initiated by separating the omentum from the greater curvature, followed by a full mobilization of the stomach and identification of the short gastric vessels. The stomach was transected using EndoGIA stapler and calibrated with a nasogastric tube, ch. 32. Prophylactic clips were applied at the resection site of the antrum. Hemostasis was ensured by additional sutures if necessary.

### Postoperative Course

At the post-anesthesia care unit (PACU), 1L Ringer lactate was administered i.v., and if necessary, postoperative pain was managed using a minimal dose of oxycodone i.v. Complete mobilization was achieved within 2 h postoperatively, prior to patients being transferred to the ward. Discharge was scheduled to postoperative day (POD) 1, following the discharge criteria from the Danish Society of Anesthesia and Intensive care Medicine (DASAIM) [13].

The standardized postoperative peroral pharmacological treatment consisted of four days of paracetamol 1 g and ibuprofen 0.4 g, both four times a day, and magnesia 1 g twice a day, and if necessary, oxycodone 5 mg, maximum six times per day. Furthermore, patients received pantoprazole 40 mg twice a day until the end of postoperative month (POM) 3.

During the first and second postoperative week, patients were contacted telephonically by a nurse and dietitian. Moreover, patients were convened to a follow-up appointment with a dietitian at POM 4, 12, and 24. Patients could contact the hospital at any time in between these appointments, including emergency contact.

### Data collection, Validation, and Definition

Data was collected prospectively, then extracted from the digital clinical record system and manually transferred to a Research Electronic Data Capture system (REDCap). Data was validated by comparison with the data from the Danish National Bariatric Registry, which is crosschecked with the Danish National Patient Registry [14, 15].

Baseline hypertension, pulmonary diseases, and psychiatric disorders were defined as pharmacologically treated. Diabetes type 2 was defined as previously diagnosed, medically treated, or HbA1c ≥ 48 mmol/mol. Prediabetes accounted for HbA1c 39–47.9 mmol/mol [16]. Dyslipidemia was defined as medically treated, HDL < 1 and/or LDL > 3 at baseline. GERD at baseline was registered if patients reported at least one of two cardinal symptoms: pyrosis, defined as a retrosternal burning sensation that ascends toward the neck, and regurgitation, defined as a passive appearance of gastric and/or intestinal content in the mouth. GERD was then categorized by medication. There was missing data on few comorbidities, varying from 0.06 to 3.3%, and 0.5% on both lifestyle parameters.

Primary outcomes were length of hospital stay (LOS), total surgery time, mortality, readmissions, reoperations as well as 30- and 90-day complications. Complications were classified using the CDC, thus defined as any deviation from the normal postoperative course needing intervention [8]. Each patient experiencing a complication was assigned one CDC grade, corresponding to the most severe intervention needed. If a patient experienced more than one complication, only the most severe was registered.

Gastric edema was defined as dysphagia with or without dehydration, nausea, and vomiting. Readmission was defined as patients staying at a hospital overnight after being discharged from hospital. Surgery time was defined from insertion of Veress cannula to application of last clamp or suture in the skin.

Secondary outcomes were weight loss and quality of life (QoL). Weight loss was analyzed at POM 4 and 12, measured as BMI (kg/m^2^), percentage total weight loss (TWL) and excess weight loss (EWL) [17]. QoL was analyzed using Moorehead–Ardelt questionnaires provided preoperatively and at POM 12 [18].

### Statistical Analysis

IBM SPSS Statistics for Mac (version 28.0, Chicago, IL, USA) was used for statistical analysis. Continuous data are presented as median and 5–95 percentiles, and categorical data as number and percentage. Statistical significance was defined as *p* < 0.05 and calculated using Friedman’s test for repeated measurements.

### Approvals

This study is approved by the Danish Data Protection Agency (R-22009044). In accordance with Danish legislation, data obtained from clinical registries can be considered quality assurance data and can thus be applied in observational studies without approval from the Danish Scientific Ethics Committee.

## Results

### Study Cohort

A total of 1600 consecutive primary SG operations were performed during 2020 and 2021. Baseline characteristics are presented in Table 1.

**Table 1:** Baseline characteristics

	*n* = 1600
Characteristics	
Sex, female	1385 (86.6%)
Age, years	41 (24; 60)
BMI at surgery day	40.3 (33.5; 52)
Prev. abdominal surgery	684 (42.8%)
Comorbidities	
Diabetes, total	86 (5.4%)
Diabetes, type 1	4 (0.3%)
Diabetes, type 2	82 (5.1%)
Prediabetes	386 (24.5%)
Hypertension	277 (17.3%)
Cardiovascular diseases	54 (3.4%)
Arrythmia	27 (1.7%)
Ischemic heart disease	13 (0.8%)
Heart failure	3 (0.2%)
Other	16 (1.0%)
Dyslipidemia, total	927 (57.9%)
Medically treated	99 (6.2%)
Endocrine diseases, total	172 (10.8%)
PCOS	73 (5.3%) *
Hypothyroidism	97 (6.1%)
Hyperthyroidism	5 (0.3%)
Other	6 (0.4%)
GERD, total	297 (18.6%)
Medically treated	157 (9.8%)
PPI treatment	143 (8.9%)
Pulmonary diseases	130 (8.1%)
Asthma or COPD	119 (7.4%)
Other	11 (0.7%)
Sleep apnea	58 (3.6%)
Psychiatric disorder	271 (16.9%)
Lifestyle	
Alcohol, units/week	
< 7	1520 (95%)
7–14	56 (3.5%)
> 14	14 (0.9%)
Smoking status	
Never smoked	820 (51.2%)
Active smoker	207 (12.9%)
Former smoker	566 (35.4%)

Data is presented as n (%) or median (5–95 percentile. *BMI*, body mass index (kg/m^2^); *PCOS*, polycystic ovarian syndrome; *GERD*, gastro-esophageal reflux disease; *PPI*, proton pump inhibitor; *COPD*, chronic obstructive pulmonary disease. *Percentage of women

### Primary Outcomes

#### Mortality, Reoperations, and Readmissions

There was no mortality within 90 days postoperatively.

Nineteen reoperations were performed within POD 30 (1.2%). Of these, 17 (89.5%) were due to bleeding, one (5.3%) was due to a port site infection, and one (5.3%) was a diagnostic laparoscopy. Fourteen (73.7%) of all reoperations happened prior to discharge from primary admission. There were no additional reoperations between POD 30 and 90.

Sixteen patients (1%) were readmitted within POD 30. The causes of readmission were six cases of internal bleeding, five cases of gastric edema, two cases of dehydration, two port site infections, and one case of nausea/vomiting. Each of these required intervention for their complications and are thus represented in Table 2. Between POD 30 and 90, four additional patients were readmitted, resulting in an overall 90-day readmission rate of 1.3%.

**Table 2:** Thirty-day complications graded by the Clavien-Dindo Classification

	*n* = 1600						
Complication	I	II	IIIa	IIIb	IV + V	***Total***	**%**
Bleeding (internal)	-	1	-	17	-	***18***	***1.1***
Gastric edema	-	17	-	-	-	***17***	***1.1***
Nausea/vomiting	14	-	-	-	-	***14***	***0.9***
Port site infection	-	5	1	1	-	***7***	***0.4***
Port site bleeding *	4	2	1	-	-	***7***	***0.4***
Dehydration	5	-	-	-	-	***5***	***0.3***
Cardiopulmonary **	2	2	-	-	-	***4***	***0.3***
Constipation	2	-	-	-	-	***2***	***0.1***
***Total***	***27***	***27***	***2***	***18***	*-*	***74***	***4.6***
***%***	***1.7***	***1.7***	***0.1***	***1.1***	*-*		

^*^Includes bleeding and hematomas

^**^Includes AFLI, pneumonia, atelectasis, and hypertensive lung stasis

#### Surgery Time and LOS

The median total surgery time was 30 min (23–43). At POD 1, 98.9% of all patients were discharged. The remaining patients were discharged on either the day of surgery (0.13%), POD 2 (0.9%) or POD 3 (0.06%). The main reason for prolonged stay was reoperation due to internal bleeding (0.6%).

#### Thirty-Day Complications

Thirty-day complications were reported in 74 patients (4.6%). There were no perioperative complications, and 22 (29.7%) of the complications occurred before discharge from primary admission. Specification of 30-day complications and CDC grades assigned are presented in Table 2*.*

#### Ninety-Day Complications

There were 13 additional complications between POD 30 and 90, resulting in a total of 87 (5.4%) complications within 90 days. Classified by CDC, 65 of these complications were ≤ grade II (74.7%) and 22 corresponded to grade III (25.3%).

### Secondary Outcomes

#### Weight Loss

**Fig. 1:**
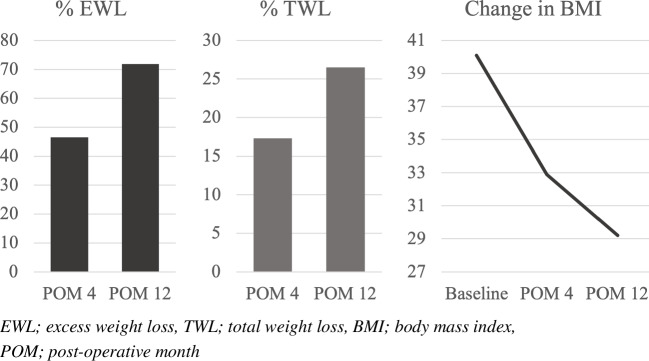
Weight loss over time

Weight data was available in 339 patients (21.2%). Weight loss was significant at each time of measurement for (*p* < 0.001). Data on BMI, EWL, and TWL are presented in Table 3 and Fig. 1.

**Table 3:** Weight loss over time

	*n* = 339
BMI (kg/m^2^)	
Baseline	40.1 (33.3; 50.7)
POM 4	32.9 (26.3; 43)
POM 12	29.2 (23.2; 38.4)
EWL (%)	
POM 4	46.5 (25.9; 84.8)
POM 12	71.9 (34.2; 115.2)
TWL (%)	
POM 4	17.3 (10.7; 26.5)
POM 12	26.5 (13.3; 40.3)

Data is presented as median and (5–95 percentiles). *BMI*, body mass index; *POM*, post-operative month; *EWL*, excess weight loss; *TWL*, total weight loss

#### Quality of Life

Data on QoL was available in 208 patients (13%). The median preoperative score was − 0.2 (− 1.8; 1.4), while the median postoperative score was 1.4 (− 0.3; 2.6). The median difference was 1.53 (− 0.3; 3.1), which was statistically significant (*p* < 0.001).

## Discussion

Fast-track has been implemented in numerous surgical fields with great improvements on postoperative recovery and overall morbidity [1, 2]. However, findings in bariatric surgery have been inconsistent [3–6]*.* This study documents ERABS principles to be highly feasible in bariatric surgery, as 99.1% of all patients were discharged within POD 1, without compromising safety and efficacy, as discussed in the following.

### Complications

Previously, complications have often been reported as reoperations, readmissions, and mortality. In this context, we had low complication rates, as mortality was zero, total 30-day reoperation rate was 1.2%, and total 30- and 90-day readmission rates were 1% and 1.3%, respectively [4, 19–21]. However, reporting complications in this manner is rather simplified and might result in an underestimation of minor complications.

The Clavien-Dindo Classification (CDC) facilitates a standardized way of reporting complications, defining complications as any deviation from the normal postoperative course, thus assuring that complications of lesser severity is reported [8, 9]. Standardization precipitates direct comparison, however, a prerequisite for this is that all complications are consistently reported. Since the postoperative course as well as the choice of intervention is not standardized between centers, CDC could still lead to a skewed reporting of complications between studies. For instance, our institution has a very low threshold for reoperation (grade III) for suspected internal bleeding, while other centers might have a different approach, e.g., blood transfusions (grade II). Furthermore, not all studies define the normal postoperative course, making comparison of minor complications challenging.

Bearing this in mind, a general comparison of our results with other reports reveals our complication rates to be considerably low, especially considering grades III–V (Table 4) [22–30]. We did not identify any studies reporting complications after SG classified by CDC, neither in an ERABS-setting, nor in a fast-track setting.

**Table 4:** Overview on CDC grades for 30-day complications from other studies on LSG

	Year(s), all included	Patients (*n*)	I (%)	II (%)	III (%)	***IIIa (%)***	***IIIb (%)***	IV (%)	V (%)	***Total*** ***(%)***
**Present study**	**2020–2021**	**1600**	**1.7**	**1.7**	**1.3**	***0.1***	***1.1***	**0**	**0**	***4.6***
Vidal et al. [22]	2004–2011	114	0	4.4	3.5	*0*	*3.5*	0	0.9	***8.8***
Peterli et al. [25]	2007–2011	107	4.7	2.8	0.9	*n/a*	*n/a*	0	0	***8.4***
Lorente et al. [26]	2008–2012	113	3.5	2.7	0.9	*0.9*	*0*	0	0.9	***8.0***
Goitein et al. [27]	2007–2014	2651	0.7	1.6	1.5	*0.7*	*0.8*	0.3	0.04	***4.1***
Falk et al. [23]	2011–2014	209	3.8	9.6	1.9	*1.0*	*1.0*	0	0	***15.3***
Lemanu et al. [24]	2007–2010	400	5.0	4.5	5.8	*n/a*	*n/a*	1.3	0.3	***16.9***
Luppi et al.* [28]	2008–2013	102	5.9	6.9	4.9	*2.0*	*2.9*	0	0	***17.7***
Głuszyńska et al. [29]	2012–2020	610	0.2	2.1	2.1	*n/a*	*n/a*	1.0	0.3	***5.7***
Husein et al. [30]	2011–2015	381	0.5	2.9	1.3	*n/a*	*n/a*	0.5	0.3	***5.5***
***Mean (present study included)***			***2.6***	***3.9***	***2.4***	***0.8***	***1.6***	***0.3***	***0.3***	***9.5***

*CDC*, Clavien–Dindo classification. *group < 60 years

Surprisingly, we could not identify any studies describing gastric edema as a complication to bariatric surgery. The lacking literature might be caused by underreporting, a general unfamiliarity, or a higher frequency among our patients, as we use a 32F tube for calibration of the sleeve, which is smaller than in other studies [22, 23, 25, 28]. However, for other esophago-gastric procedures, e.g., fundoplication, gastric edema is a well-known complication [31]. It could be an interesting aspect for further assessment in future studies.

### Weight Loss and QoL

As expected, the procedure resulted in a substantial weight loss with an EWL of 71.9% during the first postoperative year. These results are highly comparable with the literature [19, 32, 33]. For QoL, our median difference between baseline and 1 year postoperatively reflects results highly comparable to a recent, large nationwide study of bariatric surgery in Denmark [34].

### Strengths and Limitations

This study has some limitations. Firstly, due to the observational design, there is a possibility of unidentified confounders. Secondly, there was missing data on weight loss and QoL due to the time of data extraction. However, the patients with obtainable follow-up data had similar baseline BMI and QoL compared with the total cohort and was thus considered a representative group for analysis.

This study has several strengths. Data was prospectively registered, minimizing the risk of recall bias. Secondly, the large cohort minimizes the risk of overlooking rare complications. Furthermore, our institution is a single center, consisting of relatively few health care professionals handling a high volume of patients. This facilitates a certain amount of recursiveness, which is a crucial part of effective standardization. Although this is a strength, it can also be considered a limitation, as a generalization of the outcomes reported might not be applicable to other settings, e.g., centers with a low volume or interchanging staff.

## Conclusion

This study demonstrates that fast-track programs such as ERABS are highly feasible in bariatric surgery, as 99.1% of all patients were discharged within POD 1, without compromising neither safety nor efficacy of the procedure. Complication rates were considerably low compared with the available literature, and the procedure resulted in significant weight loss during the first postoperative year. This study thus provides strong arguments for a substantial benefit of implementation of ERABS principles in bariatric surgery.

## Data Availability

The data presented in this study are available on reasonable request from the corresponding author.
